# Effects of non-invasive brain stimulation on motor function after spinal cord injury: a systematic review and meta-analysis

**DOI:** 10.1186/s12984-023-01129-4

**Published:** 2023-01-12

**Authors:** Jian-Min Chen, Xiao-Lu Li, Qin-He Pan, Ye Yang, Sen-Ming Xu, Jian-Wen Xu

**Affiliations:** 1 Department of Rehabilitation Medicine, The First Affiliated Hospital of Guangxi Medical University, Guangxi, China; 2 Department of Rehabilitation Medicine, The First Affiliated Hospital of Fujian Medical University, Fuzhou, China

**Keywords:** Spinal cord injuries, Motor function, Non-invasive brain stimulation, Repetitive transcranial magnetic stimulation, Transcranial direct current stimulation

## Abstract

**Background:**

In recent years, non-invasive brain stimulation (NIBS) has been used for motor function recovery. However, the effects of NIBS in populations with spinal cord injury (SCI) remain unclear. This study aims to conduct a meta-analysis of the existing evidence on the effects and safety of NIBS against sham groups for motor dysfunction after SCI to provide a reference for clinical decision-making.

**Methods:**

Two investigators systematically screened English articles from PubMed, MEDLINE, Embase, and Cochrane Library for prospective randomized controlled trials regarding the effects of NIBS in motor function recovery after SCI. Studies with at least three sessions of NIBS were included. We assessed the methodological quality of the selected studies using the evidence-based Cochrane Collaboration’s tool. A meta-analysis was performed by pooling the standardized mean difference (SMD) with 95% confidence intervals (CI).

**Results:**

A total of 14 randomized control trials involving 225 participants were included. Nine studies used repetitive transcranial magnetic stimulation (rTMS) and five studies used transcranial direct current stimulation (tDCS). The meta-analysis showed that NIBS could improve the lower extremity strength (SMD = 0.58, 95% CI = 0.02–1.14,* P* = 0.004), balance (SMD = 0.64, 95% CI = 0.05–1.24, *P* = 0.03), and decrease the spasticity (SMD = − 0.64, 95% CI = − 1.20 to − 0.03, *P* = 0.04). However, the motor ability of the upper extremity in the NIBS groups was not statistically significant compared with those in the control groups (upper-extremity strength: *P* = 0.97; function: *P* = 0.56; and spasticity: *P* = 0.12). The functional mobility in the NIBS groups did not reach statistical significance when compared with the sham NIBS groups (sham groups). Only one patient reported seizures that occurred during stimulation, and no other types of serious adverse events were reported.

**Conclusion:**

NIBS appears to positively affect the motor function of the lower extremities in SCI patients, despite the marginal P-value and the high heterogeneity. Further high-quality clinical trials are needed to support or refute the use and optimize the stimulation parameters of NIBS in clinical practice.

**Supplementary Information:**

The online version contains supplementary material available at 10.1186/s12984-023-01129-4.

## Introduction

Spinal cord injury (SCI) refers to damage and the severe loss of neurons in the spinal cord [1], resulting in sensory, motor, or autonomic dysfunction [2]. Since SCI is incomplete in most cases, increasing the connectivity of the descending corticospinal pathway [3] and the neuroplasticity of neurons in the motor cortex to the spinal cord can benefit the restoration of motor function [4]. However, spontaneous recovery after spinal cord injury is variable and usually unsatisfactory. Standard pharmacological and rehabilitative approaches have been reported to promote the recovery of motor function, but the overall effects remain limited and vary largely among individuals [5]. Some new approaches, such as stem cell transplantation [6] and exosome therapy [7], have been reported as having certain restorative effects on the nerves, but most such therapies are invasive or are in the animal testing stage, limiting their clinical application.

In recent years, non-invasive brain stimulation (NIBS), including repetitive transcranial magnetic stimulation (rTMS) and transcranial direct current stimulation (tDCS), has received extensive attention. NIBS mainly regulates the excitability of the cerebral cortex through electric or magnetic fields, which has the potential to be a non-invasive and relatively simple means of treatment [8]. In rTMS, the time-varying magnetic field acts on the cerebral cortex to produce induced currents, affecting the brain’s metabolism in specific brain networks. In general, high-frequency stimulation (5 Hz or higher) increases cortical excitability, while low-frequency stimulation (1 Hz or below) decreases cortical excitability [9]. Moreover, a patterned form of rTMS called theta-burst stimulation (TBS) consisting of three pulses at 50 Hz and repeated at 5 Hz to reach a total number of 600 pulses, has also been extensively used [9]. Furthermore, tDCS uses a weak direct current to modulate the activity of neurons in the cerebral cortex [8]. Anodal tDCS increases the excitability of the cortex, while cathodal tDCS decreases it [9].

There has been increasing interest in investigating the potential of NIBS in improving motor function after SCI. Several reviews on the effects of NIBS in SCI have indicated that the presumed neural excitability modulation comprising these two NIBS techniques may effectively improve motor function. Despite some studies that have shown the positive effects of NIBS on motor function after SCI [10, 11], inconsistent results persist [11–14]. In addition, several high-quality randomized controlled trials (RCTs) have been published in the last few years [10, 15]. The transient effect of single-session NIBS suggests that multiple sessions may be needed to achieve persistent effects [16, 17]. Therefore, this review aims to quantitatively investigate the effects of and update the evidence regarding NIBS on motor dysfunction by evaluating all existing published RCTs with multiple sessions involving people with SCI.

## Methods

A preplanned protocol was registered in the International Prospective Register of Systematic Reviews (PROSPERO, CRD42016050444) by the recommendations of the Cochrane Handbook for Systematic Reviews of Interventions [18]. Furthermore, our review is described based on the Preferred Reporting Items for Systematic Reviews and Meta-Analyses (PRISMA) guidelines [19].

### Search strategy

Randomized control trials were identified by searching MEDLINE, EMBASE, Cochrane Central Register of Controlled Trials, and the clinical trials registry and database of the U.S. National Institutes of Health (ClinicalTrials.gov) on December 10, 2021. We put no restrictions on the year of publication in our search. Only studies published in English with the full text available were included. The following broad search terms were used: “spinal cord injuries”, “non-invasive brain stimulation”, “NIBS”, “transcranial direct current stimulation”, “tDCS”, “transcranial magnetic stimulation”,” TMS” and a string of predetermined words, which yielded a high sensibility in the search for randomized controlled trials. Search strategies were developed for each database using both free-text terms and the controlled vocabulary (MeSH and Emtree). The PubMed search strategy is illustrated in Additional file 1. A manual search was also conducted from the reference lists of previous systematic reviews to identify additional relevant studies.

### Selection criteria

The study participants included patients with motor dysfunction after SCI, meeting the diagnostic criteria for spinal cord injury (International standards for neurological classification of spinal cord injury, revised 2019). The intervention included NIBS (minimum 3 sessions with stimulation), including tDCS and rTMS. The sham NIBS groups were used as a comparator to evaluate the NIBS effects on recovering motor function. Studies were excluded if they were reviews or commentaries, basic experiments, a summary of meetings, book chapters, case reports, full text is not available, unpublished, or duplicate literature, including a sample with mixed neurologic conditions (neuropathic pain, neurogenic bladder, and so on).

### Study selection

First, two reviewers (JMC and XLL) independently screened all records based on the titles and abstracts. Second, the full texts of the primarily selected studies was subsequently retrieved and further examined carefully. All duplicate documents were removed by using Endnote (version X8). The full text of all relevant studies was subsequently retrieved and further examined carefully. The reviewers attempted consensus to establish which studies fulfilled the eligibility criteria. Any disagreements were resolved by discussion with a third senior reviewer (YY).

### Data extraction

Two reviewers independently conducted data extraction using a predefined data extraction form. Disagreements were resolved through discussion or, if required, adjudication by a third reviewer. The following variables were extracted from studies: (1) the general characteristics including authors, year of publication, (2) study designs, (3) sample characteristics including sample size, age, duration, SCI degree, level, etc., (4) interventions and control protocol type, (5) outcomes of motor function, (6) adverse effect. The mean scores and SD of the outcomes before and after the interventions were extracted, as well as the mean change scores and SD for meta-analyses. If the data reported in articles could not be used for data pooling, the authors of the articles were contacted for requesting the necessary data. Otherwise, the publications with unavailable data were removed.

### Methodological quality assessment

The quality of the included studies was evaluated using RevMan software (version 5.4, Cochrane Collaboration, Oxford, United Kingdom). All included studies were evaluated by seven domains: (1) Random sequence generation (2) allocation concealment (3) blinding of participants (4) blinding of outcome assessment (5) inadequate outcome data (6) whether selecting to report outcome (7) other possible bias. According to Cochrane Collaboration’s tool for assessing the Risk of Bias [8, 18], each domain was classified as a high, low, or unclear risk of bias. Studies with a low risk of bias in three or more domains were suggested as trials of moderate to high methodological quality [8]. Usually, tests for funnel plot asymmetry are performed only when at least 10 studies are included in a meta-analysis [18]. Although 14 studies were included in this analysis, when sorted by outcomes, each outcome contained fewer than 10 studies. Thus, publication bias in these trials could not be assessed by graphical analysis of the funnel plot [18].

### Outcome indicators

First, data were divided into several meta-analyses to identify possible NBIS effects. The primary outcomes were functional level, extremity strength, mobility, spasticity, and balance of each study (Table 1). When multiple outcome measures were reported without indication of a primary outcome, representative measures in the area of SCI research were chosen based on their validity and reliability [20]. We pooled the data using the change of the outcomes, if available. If not, they were estimated from the final and baseline values. Outcome measures chosen by these criteria are summarized in Table 1. The extremity strength was measured by the American Spinal Injuries Association (ASIA) impairment scale Upper Extremity Motor Score (UEMS) and Lower Extremity Motor Score (LEMS). The upper extremity function was measured by Jebsen Taylor hand function test (JTHFT). The mobility was measured as the 10 min walking test (10MWT), 6-min walking test (6MWT), and timed up and go test (TUG) respectively. The spasticity was measured using the upper/ lower Modified Ashworth Scale (MAS) and Hmax/Mmax amplitude ratio (H/M). Body balance was measured by Berg Balance Test (BBT). Subsequently, subgroup analyses based on the mean post-injury time were performed to identify the potential differences in primary outcome parameters between the subacute and chronic stages. Data from crossover studies were considered taking into account the two periods of the study to warrant a correct analysis of crossover studies and reduce bias [18].

**Table 1:** Sample characteristics, study design, and outcome measures used in the included NIBS studies

Study, year	Country	Design	Sample	Participants Mean (SD)	Relevant outcome Measures (meta-analysis)	Follow-up
[22] Tolmacheva et al. 2017	Finland	Parallel Randomized Double-blind Sham-controlled	n = 5 ASIA Scale (A/B/C/D): B1C3D1 Level of injury (C/T/L): C5 Gender (male): 4 Etiology: –	Age (years): 47.8 (12.85) Duration (months): 51.8 (25.88)	Spasticity = UMAS, LMAS Timing; 0, 4 week	1 month
[25] Benito et al. 2012	Spain	Crossover Randomized Double-blind Sham-controlled Washout period: 3-weeks	n = 17 ASIA Scale (A/B/C/D): D17 Level of injury(C/T/L): C7T10 Gender (male): 13 Aetiology: Traumatic 9; non-traumatic 8	Age (years): 38.10 (13.49) Duration (months): 7.88 (3.12)	Extremity strength = LEMS Extremity function = JTHFT Mobility function = 10MWT Spasticity = LMAS Balance = TUG Timing;0, 3 week	2 weeks
[26] Gharooni et al. 2018	UK	Crossover Randomized Single-blind Sham-controlled Washout period:2-weeks	n = 10 ASIA Scale (A/B/C/D): B1C4D5 Level of injury(C/T/L): C10 Gender(male): 8 Aetiology: Traumatic 8; non-traumatic 2	Age(years):46.8 (12.5) Duration(months):11.4(14.96)	Extremity strength = UEMS, LEMS Spasticity = UMAS Timing;0, 4 week	–
[27] Gomes-Osman et al. 2015	USA	Crossover Double-blind Randomized Sham-controlled	n = 11 ASIA Scale (A/B/C/D): C5D6 Level of injury(C/T/L): C11 Gender(male): 1 Etiology: Traumatic 11	Age (years): 46.7(12) Duration (years):6.6 (8.2)	Extremity function = JTHFT Timing; before and after the intervention	–
[10] Krogh et al. 2022	Denmark	Double-blinded, Randomized Sham-controlled	n = 19(10/9) ASIA Scale (A/B/C/D); Exp = A1C3D6; Con = C2D7; Level of injury(C/T/L): Exp = C5T4L1; Con = C6T1L2; Gender(male): Exp = :8; Con = 7 Aetiology: Exp = traumatic 3; non-traumatic 7 Con = traumatic 3; non-traumatic 6	Age (years): Exp = 57.1 (8.3); Con = 51.8 (12.1) Duration (days): Exp = 91.3(40.8); Con = 87.3(69.5)	Extremity strength = LEMS Mobility function = 6MWT,10MWT Balance = TUG Timing; 0, 4 week	
[23] Kumru et al. 2016	Spain	Double-blind Randomized Sham-controlled	n = 31 (15/16) ASIA Scale (A/B/C/D): Exp = C12D3; Con = C14D2 Level of injury(C/T/L): Exp = C8T7; Con = C6T10 Gender (male): Exp = 10; Con = 14 Aetiology: Exp = traumatic 6; non-traumatic 9 Con = traumatic 8; non-traumatic 8	Age (years): Exp = 46.4(15.5); Con = 48.7 (16.5) Duration (months): Exp = 3.5(1.6); Con = 2.5(1.5)	Extremity strength = UEMS, LEMS Spasticity = LMAS Timing; 0,4 week	4 weeks
[28] Nardone et al. 2014	Austria	Crossover Double-blind Randomized Sham-controlled Washout period: 4-weeks	n = 9 ASIA Scale (A/B/C/D):C4D5 Level of injury(C/T/L):C5T4 Gender (male): 8 Aetiology: traumatic 7; non-traumatic 2	Age (years): 45.67(11.73) Duration (years):10.44(4.19)	Spasticity = LMAS, H/M amplitude ratio Timing;0, 1 week	1 week
[29] Nardone et al. 2017	Austria	Crossover, Randomized Double-blind Sham-controlled Washout period: > 2 months	n = 10 ASIA Scale (A/B/C/D): C4D6 Level of injury (C/T/L): C6T4 Gender (male): 7 Aetiology: traumatic 9; non-traumatic 1	Age (years): 42.8 (12.52) Duration (years):8.3 (4.79)	Spasticity = LMAS, H/M amplitude ratio Timing; 0, 2 week	1 week, 4 weeks
[30] Kumru et al. 2010	Spain	Crossover Randomized Double-blind Sham-controlled Washout period: 2-weeks	n = 15 ASIA Scale (A/B/C/D): C10D5 Level of injury(C/T/L): C4T11; Gender(male):13 Aetiology: traumatic 10; non-traumatic 5	Age (years): 36.2 (15.8); Duration (months):7.3 (3.9)	Spasticity = LMAS, H/M amplitude ratio Timing; 0, 4 week	2 weeks
[12] Kumru et al. 2016	Spain	Randomized Double-blind Sham-controlled	n = 24(12/12) ASIA Scale (A/B/C/D): Exp = C11D1; Con = C9D3 Level of injury(C/T/L): Exp = C7T5; Con = C8T4 Gender(male): Exp = :8; Con = 8 Aetiology: Exp = traumatic 8; non-traumatic 4 Con = traumatic 5; non-traumatic 7	Age (years): Exp = 49.67 (15.05); Con = 52.83 (13.91) Duration (months): Exp = 3.42(1.56); Con = 4.92 (2.13)	Extremity strength = LEMS Timing;0, 4 week	1 month
[24] Raithatha et al. 2016	USA	Randomized, Double-blind Sham-controlled	n = 15 (9/6) ASIA Scale (A/B/C/D): Exp = C7D1B1; Con = C4D2 Level of injury (C/T/L): Exp = C4T4L1; Con = C5L1 Gender (male): Exp = 5; Con = 5 Aetiology: traumatic 15	Age (years): Exp = 40.56 (12.24); Con = 58.0 (5.36) Duration (years): Exp = 8 (8.43); Con = 7.67 (15.36)	Mobility function = 10MWT, 6MWT Balance = TUG, BBT Timing; 0, 12 week	4 weeks
[15] Simis et al. 2021	USA	Parallel Randomized Double-blind	n = 43(21/22) ASIA Scale (A/B/C/D): Exp = C10D11; Con = C10D12 Level of injury(C/T/L): Exp = C4T17; Con = C11T11 Gender(male): Exp = 17; Con = 15 Aetiology: Exp = traumatic 19; non-traumatic 2 Con = traumatic18; non-traumatic 4	Age (years): Exp = 31(14.82); Con = 41 (15.56) Duration (months): Exp = 16 (13.33); Con = 15.5 (11.85)	Mobility function = 10MWT, 6MWT Spasticity = LMAS Balance = TUG, BBT Timing; 0, 4 week	3 months
[11] Yozbatiran et al. 2016	USA	Randomized Double-blind Sham-controlled	n = 8(4/4) ASIA Scale (A/B/C/D): Exp = C1D3; Con = C2D2 Level of injury(C/T/L): Exp = C4; Con = C4 Gender (male): Exp = 4; Con = 3 Aetiology: –	Age (years): Exp = 49.7(5.40); Con = 55.7(2.90) Duration (months): Exp = 25.2(10.4); Con = 141.2(48.2)	Extremity function = JTHFT Timing;0, 2 week	2 months
[13] Potter-Baker et al. 2017	USA	Longitudinal Randomized, Double-blinded Sham-controlled	n = 8 (4/4) ASIA Scale (A/B/C/D): B2D6 Level of injury(C/T/L): C8 Gender (male): 8 Etiology: traumatic 8	Age (years): 53.3 (4.1) Duration (months): Exp = 54.4(30.8); Con = 164(153.6)	Extremity strength = UEMS Timing; 0, 2 week	3 months

*ASIA Scale* American Spinal Injury Association motor score and class, *Level of injury* C, Cervical, T, thoracic, L, lumbar, *Con* control group, *Exp* experimental group, *LEMS* ASIA lower extremity motor score, *UEMS* ASIA upper extremities motor score, *MAS* Modified Ashworth Scale, *TUG* timed up and go test, *JTHFT* Jebsen-Taylor Hand Function Test, *BBT* Berg Balance Test, *10MWT* 10-min walk test, *6MWT* 6-min walk test

### Statistical analysis

Meta-analyses were performed using RevMan software (version 5.4, Cochrane Collaboration, Oxford, United Kingdom). To combine the outcomes of the included studies, the standardized mean difference (SMD) with 95% confidence intervals (CI) was calculated with a random-effect model and weighted by the pooled effect size. P < 0.05 was considered to be statistically significant. The statistical heterogeneity between the studies was assessed using Cochran’s Q test and quantified with the I^2^ statistic (I^2^ ≥ 50% indicated substantial heterogeneity) [21]. To identify the sources of heterogeneity, sensitivity analysis was conducted. Meta-regression was also performed to explore the influence of the mean age of participants on the effectiveness of NIBS interventions. Meta-regression and sensitivity analysis was carried out using STATA software (version 16.0).

## Results

### Identification of studies

**Fig. 1:**
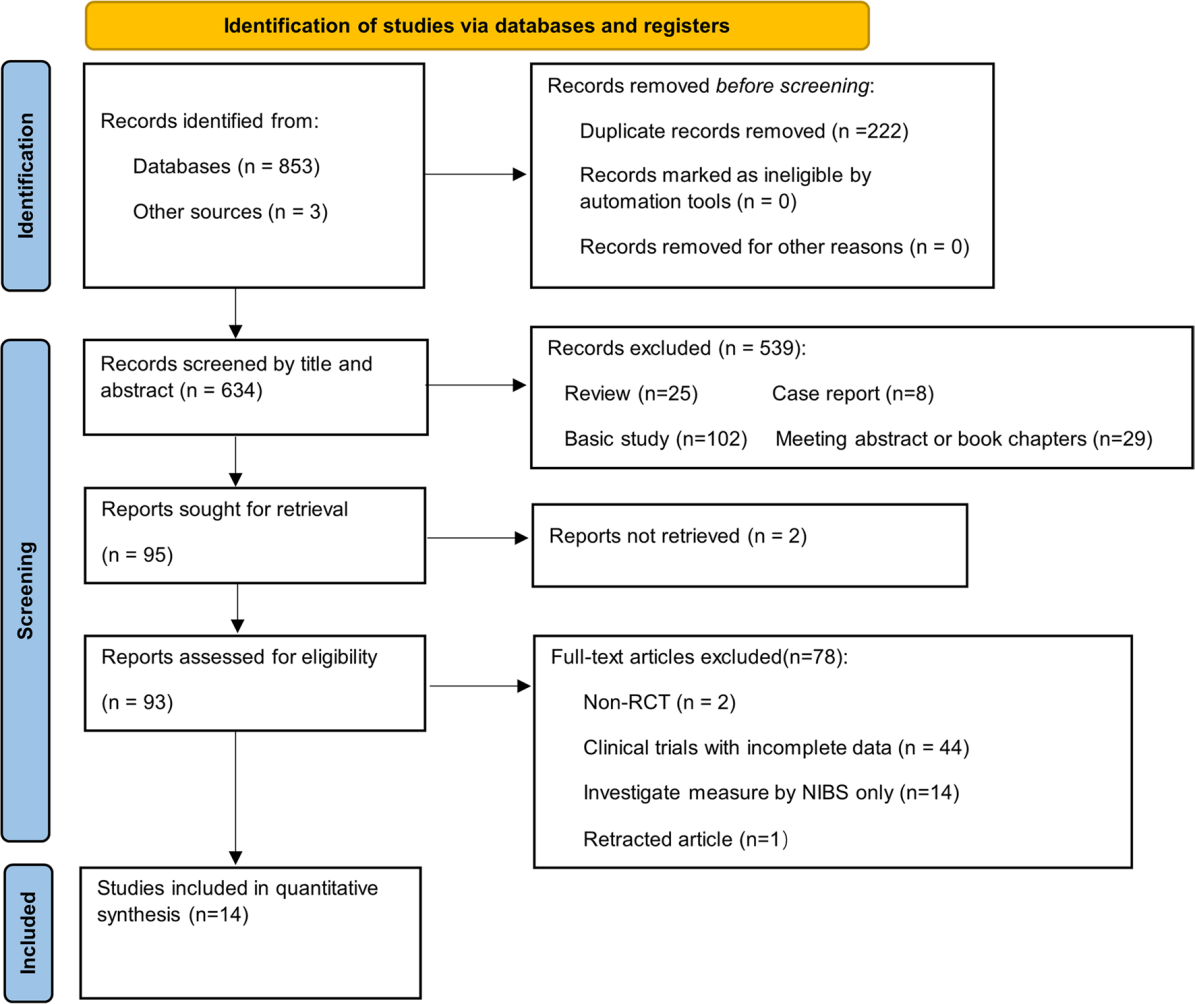
Flow of studies through the review

A PRISMA flowchart of study selection by stage of the systematic review is shown in Fig. 1. A total of 14 studies met our inclusion criteria and were described in qualitative analysis. All studies were published in English. Four trials were conducted in Spain, 4 in the U.S.A, 1 in the UK, 1 in Finland, 1 in Denmark, and 2 in Austria.

### Study selection and characteristics

Characteristics of studies are summarized in Tables 1, and 2 and summarized below. All of the included controlled studies were RCTs. Eight trials compared parallel intervention groups [10–13, 15, 22–24] and 6 studies [25–30] used a crossover design. The test groups all received NIBS, and patients in the control group received sham stimulation. In addition, 10 of the 14 total studies included in these meta-analyses used other rehabilitation therapies combined with NIBS [10–13, 15, 22–25, 27]. The form and length of these therapies for motor function were both highly variable parameters. The length ranged from one week to three months. In terms of form, 2 studies combined physical therapy [16, 27], 5 combined robot training [11, 12, 15, 23, 24], 1 combined peripheral nerve stimulation [22], 1 combined massed practice [13],1 combined antispastic medication [30]. The mean post-injury time of enrolled participants in five studies was between 1 and 12 months (subacute stage) [10, 12, 23, 25, 30], and in nine studies was greater than 12 months (chronic stage) [11, 13, 15, 22, 24, 26–29]. As regards the stimulation pattern of TMS, all but one [22] of the remaining studies used excitability stimulation patterns. All articles involving tDCS used anodal stimulation. The frequency of treatment ranged from three [24, 27] to four [22], five [10–13, 15, 23, 26, 28–30], or seven [25] times per week. The duration of treatment ranges from 3 [25] to 36 [24] sessions. The treatment intensity (in terms of session duration) ranged from 200 s [26, 29] to 30 min [13] and the treatment time did not differ between the control and treatment groups. Due to the large heterogeneity in study designs about follow-up, the first assessment available after the intervention was chosen as a follow-up. The data were sorted and analyzed based on the outcome to provide an effective evaluation of NIBS on each of the aspects: 5 studies used results from UEMS [10, 12, 23, 26] on the strength of upper extremity, 2 used JTHFF on function of the upper extremity [11, 27] and 5 used LEMS [10, 12, 23, 25, 26] on the strength of lower extremity, 2 used UMAS [22, 26], 7 used LMAS [15, 22, 23, 25, 28–30] and 3 used H/M ratio [28–30] on spasticity, 3 used BBT [15, 24, 25] on body balance, 4 used 10MWT [10, 11, 15, 24, 25], 3 used 6MWT [10, 15, 24] and 4 used TUG [15, 24] on mobility. Thus, the results of these clinical trials were pooled in different meta-analyses.

**Table 2:** Detailed NIBS setting and number/frequency of sessions used in each included study

Study	Stimulation type	Session (n)	Stimulation site	NIBS parameter	Adverse effects (n)	Rehabilitation parameters
[22] Tolmacheva et al. 2017	Exp = Active TMS + PNS Con = Sham TMS + PNS	16	Arm primary motor cortex	0.2 Hz, 20 min, 100%RMT during 4 weeks	–	PNS: 20 min/day, 5 days/week or 3 days/week for 4 weeks
[25] Benito et al. 2012	Exp = Active rTMS + Rehabilitation therapy Con = Sham rTMS + Rehabilitation therapy	15	Leg motor area	20 Hz for 2 s bursts, intertrain intervals of 28 s, over 20 min, 90%RMT, 1800 pulses/session, 15 days	–	Rehabilitation therapy: 5 h/ session, 15 sessions for 3 weeks
[26] Gharooni et al. 2018	Exp = Active iTBS Con = Sham iTBS	10	Primary motor cortex	50 Hz for 3 stimuli, 80%RMT, 600 pulses/session in 200 s for 2 weeks	–	–
[27] Gomes-Osman et al. 2015	Exp = Active TMS + RTP Con = Sham TMS + RTP	3	Corticomotor hand region	10 Hz, 80%RMT, 800 pulses/session, 3 days/week for 1 week	Transient Headache: 3	RTP:1 week
[10] Krogh et al. 2022	Exp = Active rTMS + LL-RT classes + LL-PT classes Con = Sham rTMS + LL-RT classes + LL-PT classes	20	Leg primary motor cortex	20 Hz, 100% RMT, 1800 pulses/session, over 22 min/day, 5 days/week for 4 weeks	Seizure: 1	LL-RT classes + LL-PT classes LL-RT classes: twice weekly for 4 weeks LL-PT Classes: 60 min/session, thrice weekly for 4 weeks
[23] Kumru et al. 2016	Exp = Active TMS + Lokomat training Con = ShamTMS + Lokomat training	20	Motor area	20 Hz for 2-s duration bursts, 90%RMT, 1800 pluses/session over 20 min, 5 days/week for 4 weeks	Facial muscle contraction:8 Mild headache: 1	Lokomat training:30–45 min/day, Monday to Friday/week, 8 weeks
[28] Nardone et al. 2014	Exp = Active TMS Con = Sham TMS	5	Primary motor cortex	20 Hz for 2-s long bursts, 90%RMT, 1600 pulses/session over 20 min, 5 days/week for 1 week	–	–
[29] Nardone et al. 2017	Exp = Active TMS Con = Sham TMS	10	Leg area of dominant primary motor cortex	50 Hz, 80%AMT, 600 pulses/session iTBS:3 pulses of 50 Hz repeated at 5 Hz for total of 600 stimuli (200 s), 5 days/week for 2 weeks	–	–
[30] kumru et al. 2010	Exp = Active TMS + Antispastic medication Con = Sham TMS + Antispastic medication	5	Primary motor cortex	20 Hz for 2-s duration bursts, 90%RMT, 1600 pulses/session, over 20 min, 5 days/week for 1 week	Twitching facial muscles:3	–
[12] Kumru et al. 2016	Exp = anodal tDCS + Lokomat training Con = Sham tDCS + Lokomat training	20	Anode = leg motor cortex Cathode = non-dominant supraorbital area	2 mA × 20 min/day, 5 days/week for 4 weeks	–	Lokomat training: 30 min/day, Monday to Friday/week for 8 weeks
[24] Raithatha et al. 2016	Exp = anode tDCS + Lokomat training Con = Sham tDCS + Lokomat training	36	Lower extremity motor cortex	2 mA × 20 min/day, 3 days/week for 12 weeks, current density of 0.08 mA/cm^2^	–	Lokomat training: 1 h/time, 3 times/week for 12 weeks
[15] Simis et al. 2021	Exp = Active TMS + Lokomat training Con = Sham TMS + Lokomat training	30	Primary motor cortex	2 mA × 20 min/day, 3 days/week for 10 weeks (outpatient group) or 5 days/week for 6 weeks (inpatients group)	Tingling and itching	Lokomat training: 30 min/time, 3 times per week over 12 weeks or 5 times per week over 6 weeks
[11] Yozbatiran et al. 2016	Exp = anodal tDCS + exoskeleton robot-assisted arm training Con = Sham tDCS + exoskeleton robot-assisted arm training	10	Primary motor cortex	2 mA × 20 min/day, 5 days/week for 2 week, current density of 0.0571 mA/cm^2^	Tingling, skin redness, and sleepiness	exoskeleton robot-assisted arm training: 3 h/session, 12 sessions over 4 weeks
[13] Potter-Baker et al. 2017	Exp = anodal tDCS + MP Con = Sham tDCS + MP	10	Primary motor cortex	2 mA × 30 min/day, 5 days/week for 2 weeks	–	MP: 2 h/time, 5 times/week over 2 weeks

*rTMS* repetitive transcranial magnetic stimulation, *RMT* resting motor threshold, *Con* control group, *Exp* experimental group, *iTBS* intermittent Theta-burst stimulation, *tDCS* transcranial direct current stimulation, *PNS* peripheral nerve stimulation, *RTP* repetitive task practice, *LL-RT* lower limb resistance training, *LL-PT* lower limb physical therapy, *MP* massed practice

A total of 225 subjects were included in the 14 studies. The basic characteristics of the included literature are shown in Table 1. The number of participants in each study ranged from 5 [22] to 43 [15]. The pooled sample was predominantly males (73.78%) with a mean (SD) of 44.31 (15.08) years of age and 2.77 (5.32) years of duration of post-injury. All included studies provided information on the level of spinal cord injury and baseline severity according to the ASIA. The number of patients with the injury level cervical was 128 (56.89%) of the sample, whereas thoracic was 92 (40.89%) and lumbar was 5 (2.22%). Complete SCI at A level of impairment in ASIA was present only in 1 patient, and incomplete SCI at B level was in 5(2.22%), level C in 115 (51.11%), and level D in 104 (46.22%) of these patients.

### Adverse effects

Among 14 included studies, 8 reported no obvious adverse effects [12, 13, 22, 24–26, 28, 29]. One study has reported that 1 patient experienced a seizure during TMS stimulation [10]. Five studies reported minor adverse effects [11, 15, 23, 27, 30], such as tingling, itching, skin redness, sleepiness, facial muscle contraction, or headache, which were observed also in the sham group.

### Quality

**Fig. 2:**
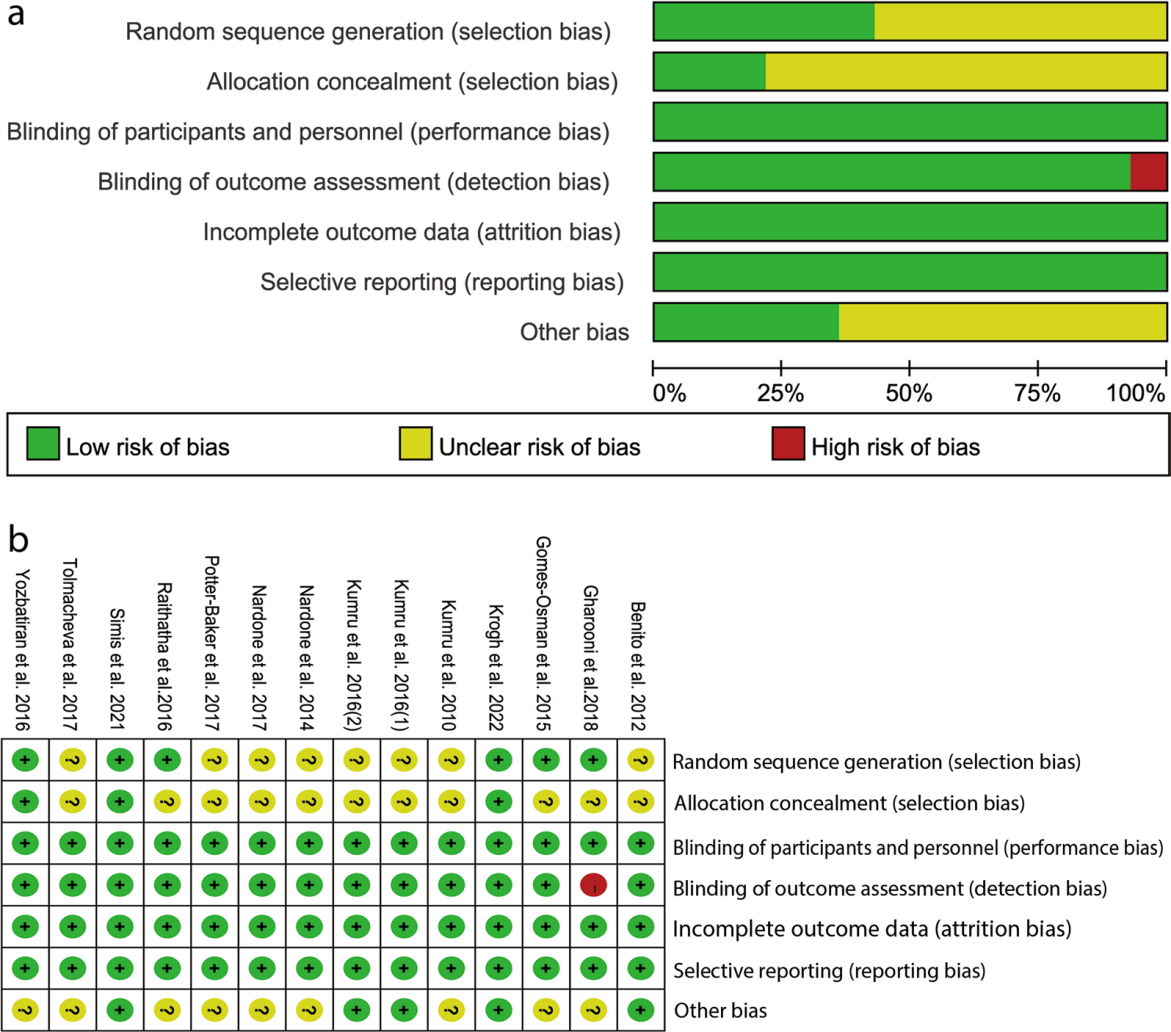
Cochrane risk of bias assessment of the included studies. **a** Risk of bias graph; **b** Risk of bias summary

Figure 2 presents the authors’ judgments about the risk of each biased domain and percentages of risks across all included studies. 8 studies (57.14%) [12, 13, 22, 23, 28–30] reported adequate random sequence generation and 3 (21.43%) [10, 11, 15] hid the allocation scheme, all presented blinding of participants and personnel, 13 (92.86%) presented blinding of outcome assessment and all described a low risk for attrition, showing a low risk of bias. Therefore, all of the included studies presented moderate to high methodological quality.

### Effects of interventions

#### Extremity strength and function

**Fig. 3:**
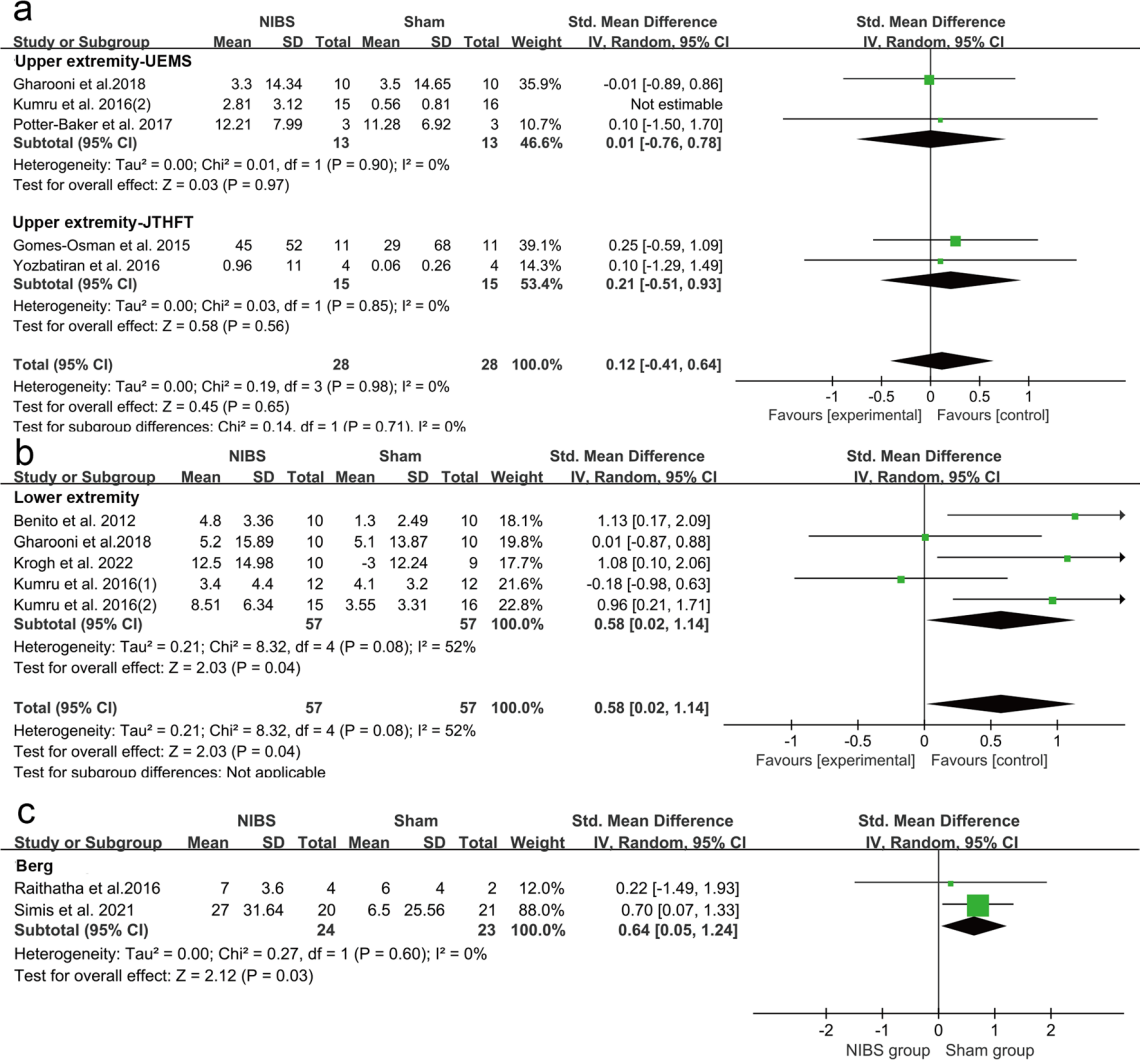
Weighted mean difference (95% CI) of the effect of NIBS compared with sham on (**a**) lower extremity strength by pooling data from 5 trials (LEMS), upper extremity strength by pooling data from 3 trials (UEMS) and upper extremity function by pooling data from 2 trails (JTHFT) in people with SCI; (**b**) Balance from 2 tails

**Fig. 4:**
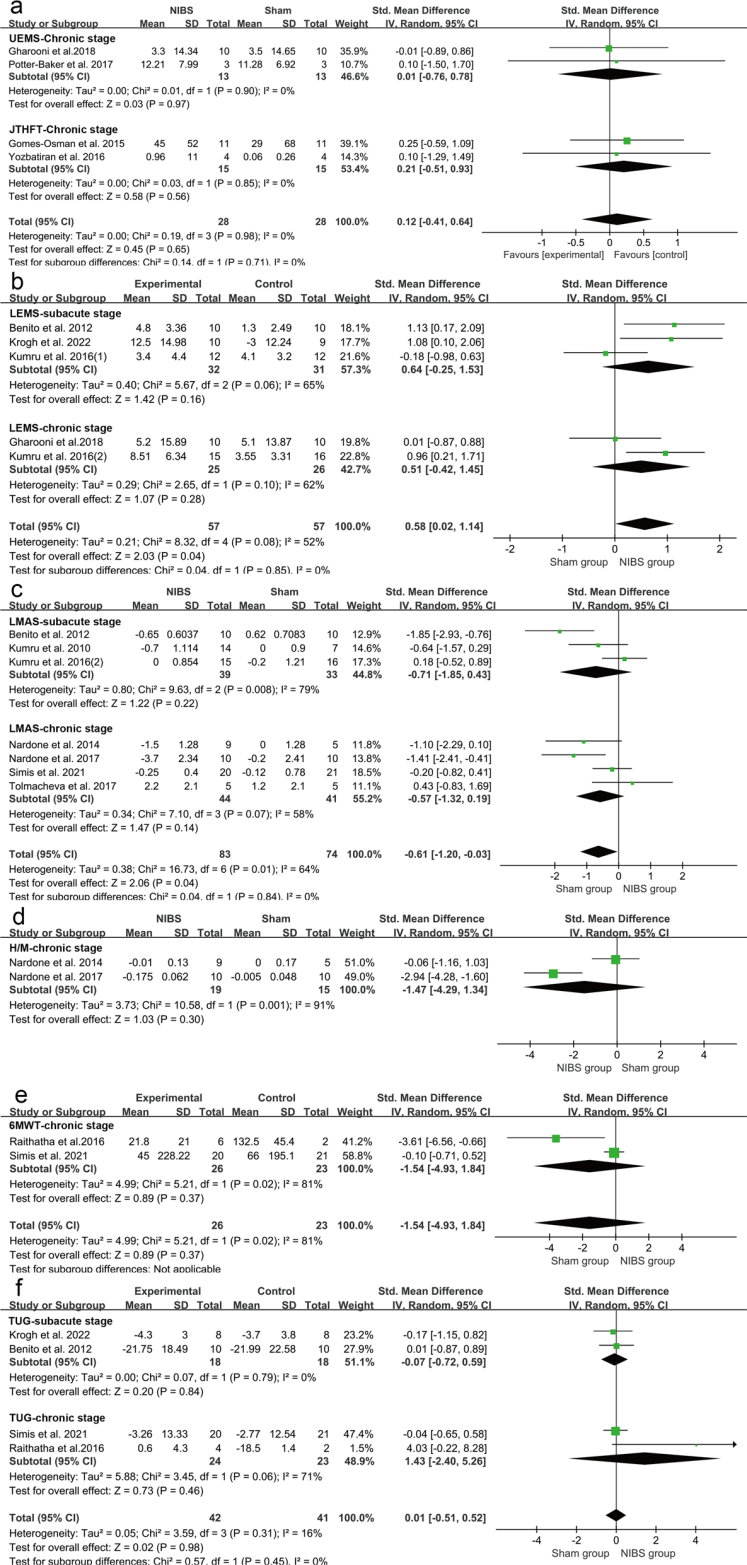
A subgroup analysis based on different SCI stages for the NIBS effects compared with sham on (**a**) upper extremity strength, (**b**) lower extremity strength, (**c**) LMAS, (**d**) H/M, (**e**) 6MWT and (10) TUG

No greater improvements in the upper extremity performance (strength and function) assessed by UEMS and JTHFT were observed in the NIBS groups compared to the sham groups (SMD = 0.12, 95% CI = − 0.41–0.64; *P* = 0.65; I^2^ = 0%) (Fig. 3a). When only studies for the chronic stage were analyzed, the pooled effect remained insignificant (SMD = 0.12, 95%CI = − 0.41–0.64, *P* = 0.65, I^2^ = 0%) (Fig. 4a). In the NIBS group, lower extremity strength measured by LEMS was greater than that in the sham groups (SMD = 0.58, 95% CI = 0.02–1.14, *P* = 0.04, I^2^ = 52%) (Fig. 3b). When the studies for sub-acute stage or chronic stage were analyzed separately, the heterogeneities were both increased and the pooled effects were no longer significant (Subacute stage: SMD = 0.64, 95%CI = − 1.25–0.53, *P* = 0.16, I^2^ = 65%; Chronic stage: SMD = 0.51. 95% = − 0.42–1.45, *P* = 0.28. I^2^ = 62%) (Fig. 4b).

#### Balance

Significant improvements by BBT were observed in the NIBS groups compared to the sham groups (SMD = 0.64, 95% CI = 0.05–1.24, *P* = 0.03, I^2^ = 0%) (Fig. 3c).

#### Spasticity

**Fig. 5:**
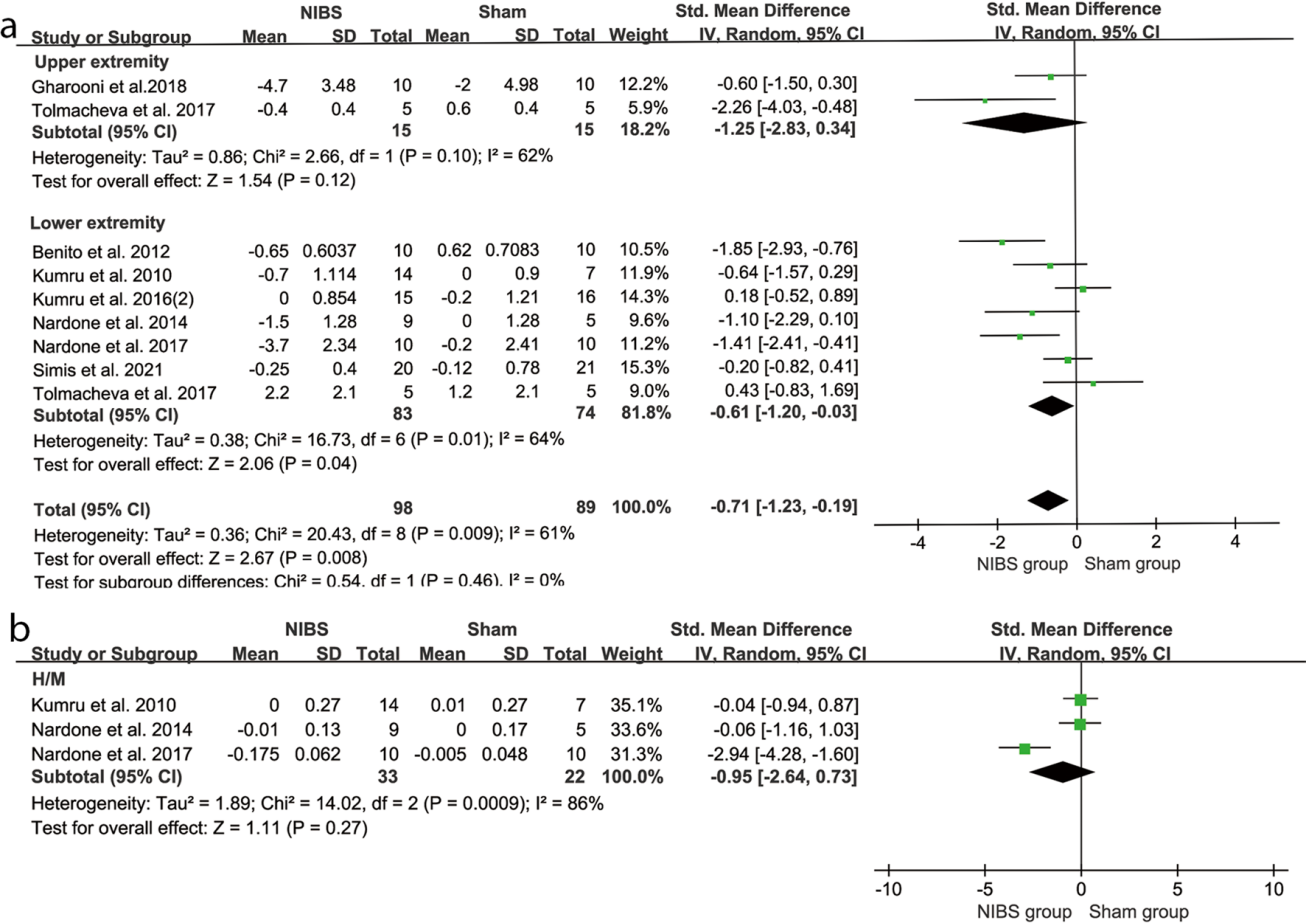
Weighted mean difference (95% CI) of the effect of NIBS compared with sham on (**a**) upper extremity spasticity by pooling data from 2 trials (UMAS) and lower extremity strength by pooling data from 7 trials (LMAS) in people with SCI; (**b**) H/M ratio from3 tails

The changes of spasticity in upper limbs assessed by UMAS were not more significant in the NIBS groups than in sham groups (SMD = − 1.25, 95% CI = − 2.83–0.34, *P* = 0.12, I^2^ = 62%) (Fig. 5a). The changes of spasticity in lower limbs assessed by LMAS were more significant in the NIBS group than those in the sham groups (SMD = − 0.61, 95% CI = − 1.20 to − 0.03, *P* = 0.04, I^2^ = 64%) (Fig. 5a). When only subacute or chronic stage studies were analyzed for LMAS, heterogeneities were slightly changed and the pooled effects were no longer significant (Subacute stage: SMD = -0.71, 95%CI = − 1.85–0.43, *P* = 0.22, I^2^ = 79%; Chronic stage: SMD = − 0.57, 95%CI = − 1.32–0.19, *P* = 0.14, I^2^ = 58%) (Fig. 4c). However, overall changes in spasticity of lower limber measured by H/M ratio were similar in the NIBS groups and sham groups (SMD = − 0.95, 95% CI = − 2.64–0.73, *P* = 0.27, I^2^ = 86%) (Fig. 5b). When only studies for chronic stage were analyzed, heterogeneity was further increased and the pooled effects were still insignificant (SMD = − 1.47, 95% CI = − 4.29–1.34, *P* = 0.30, I^2^ = 91%) (Fig. 4d).

#### Mobility

**Fig. 6:**
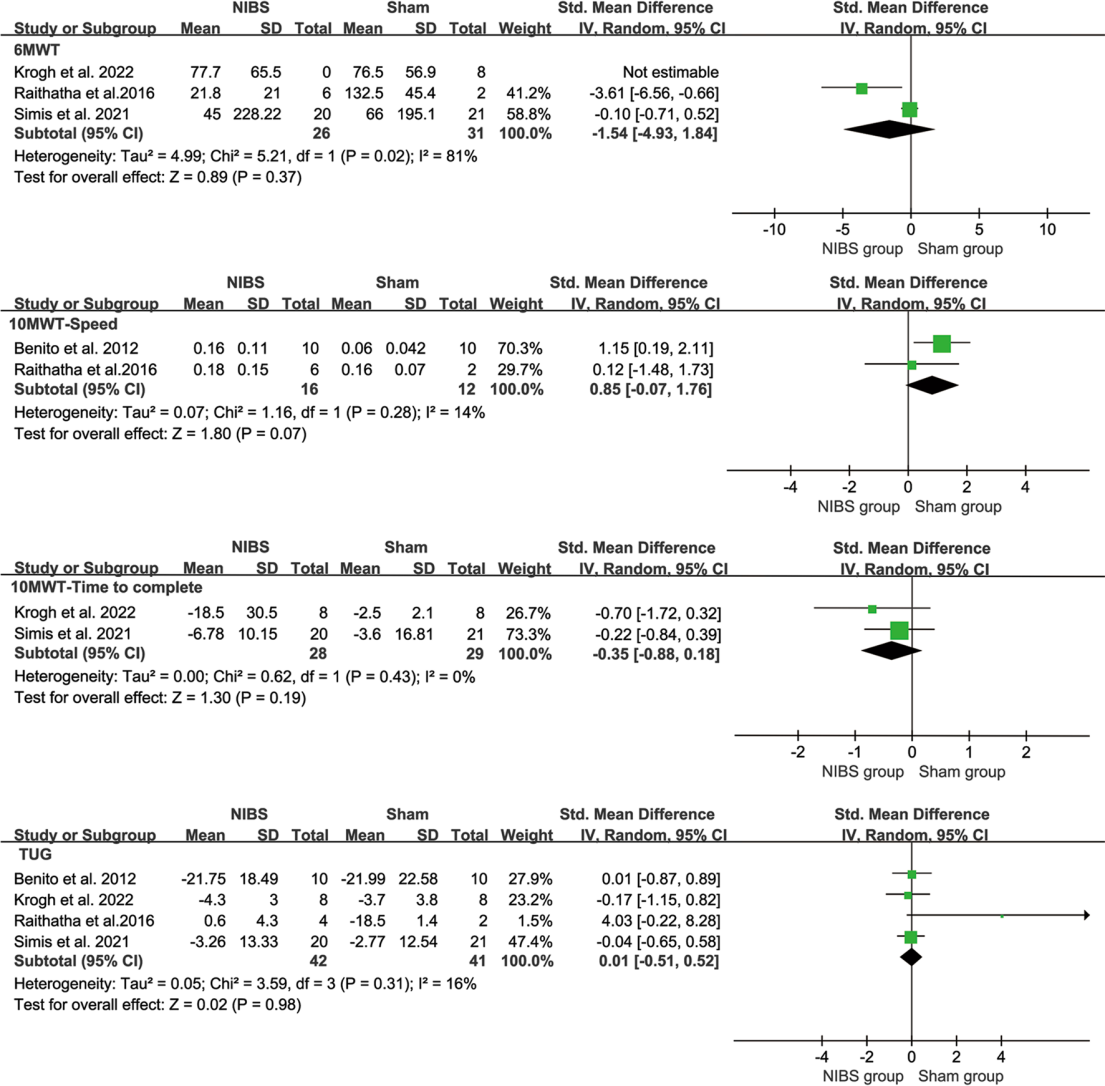
Weighted mean difference (95% CI) of the effect of NIBS compared with sham on mobility by pooling data from (**a**) 3 trials of 6MWT, (**b**) 2 trials of 10MWT in speed, (**c**) 2 trials of 10MWT in time- to-complete, (**d**) 4 trials of TUG

Mobility was similar in the NIBS groups and sham groups, which was evaluated by the gait distance of 6MWT (SMD = − 0.17, 95% CI = − 0.68–0.34; *P* = 0.51; I^2^ = 63%) (Fig. 6a), the speed of 10MWT (SMD = 0.85, 95% CI = − 0.07–1.76; *P* = 0.07; I^2^ = 14%) (Fig. 6b), time-to-complete the 10MWT (SMD = − 0.35, 95% CI = − 0.88–0.18; *P* = 0.19; I^2^ = 0%) (Fig. 6c), and TUG (SMD = 0.01, 95% CI = − 0.51–0.52, *P* = 0.98, I^2^ = 16%) (Fig. 6d). For 6MWT, when only studies for the chronic stage were analyzed, heterogeneity was further increased and the pooled effects were still insignificant (SMD = − 0.44, 95% CI = − 1.53–0.65, *P* = 0.37, I^2^ = 81%) (Fig. 4e). For TUG, the heterogeneity was decreased when only studies for the subacute stage were analyzed (I^2^ = 0%) and increased for only chronic stage studies (I^2^ = 71%). However, the pooled effects were still not significant (substage stage: SMD = − 0.07, 95% CI = − 0.72–0.59, *P* = 0.84; chronic stage: SMD = 1.43, 95% CI = − 2.40–5.26, *P* = 0.46) (Fig. 4f).

#### Meta-regression

The age of participants is a highly heterogeneous parameter. The univariate meta-regression analysis was performed to identify any association between mean age and effect size. The results showed that the mean age was not a significant predictor of the effect size. However, these analyses may be underpowered, given the small number of studies involved (much less than 10) [31, 32]. The results of the univariate meta-regression are presented in Additional file 2.

#### Sensitivity analysis results

Sensitivity analysis revealed that the heterogeneity across subgroups did not change after excluding any one study, suggesting the source of the heterogeneity was multifaceted. The results are shown in Additional file 3.

## Discussion

As a new neuromodulation technique, NIBS has been reviewed for its potential to improve motor function after SCI [3, 17]. The present systematic review and meta-analysis evaluate the effects and summarize the safety profiles of NIBS. The data for participants of the included trials in this review demonstrated evidence that NIBS has positive effects on the strength and spasticity of the lower extremities, as well as balance.

From a motor control perspective, damage to spinal tracts disturbs the information transmission from the brain to the spinal cord. In addition, the damage also results in maladaptive reorganization of the entire neuraxis, contributing to motor dysfunction [17]. At the spinal level, the maladaptive reorganization of spinal circuits leads to spasticity, due in part to the loss of descending control of inhibitory spinal circuits [33]. Clinically, injuries are divided into the categories of (neurologically) complete or incomplete, depending on the presence or absence of neurological functions below the segmental level of injury. Numerous histological analyses and electrophysiologic studies have demonstrated that most patients diagnosed with complete SCI with loss of all neurological functions below the injury have residual physiological or anatomical continuity of the central nervous system tracts across the lesion. These residual tracts provide a fertile ground for NIBS, which is concerned with establishing central axonal regeneration and reestablishing physiological reconnections [34].

NIBS is applied over the motor cortex in SCI patients to take advantage of neuroplasticity to activate the residual axon and establish functional connectivity in the corticospinal tract. Some clinical studies have indicated that the ultimate therapeutic effects are produced by affecting signaling in the nervous system; namely, by exciting, inhibiting, or regulating neuronal and neural network activities [35, 36]. In addition, several studies in animal models of SCI have also suggested the benefits of NIBS. Some studies have indicated that following injury, NIBS can enhance the spontaneous collateral or regenerative sprouting of corticospinal tracts, increase the regeneration rate of axons, as well as produce motor recovery corresponding to increased axonal growth [36, 37]. Another study showed that tDCS can increase the expression of a brain-derived neurotrophic factor in mouse cortical slices, which can promote changes in synaptic plasticity [38]. Poirrier et al. reported that after eight weeks of treatment with 10 Hz rTMS, a significant positive correlation between the final motor function of SCI in animal models and the grey matter density of the serotonergic fibers in the spinal segment [39]. Cao et al. reported that the ability of rTMS to alleviate spasticity and promote motor function following SCI might be related to the varying degrees of up-regulation of GABA receptors [40] and potassium-chloride cotransporter-2 protein [41]. However, the study by Poirrier et al. has suggested a mechanism by which rTMS is beneficial in low thoracic lesions because it activates the central locomotor generator [42].

In our meta-analysis, the motor cortex was selected as a stimulation site in all studies, and NIBS showed a significant effect on lower-extremity strength and balance. However, there was no greater improvement in functional mobility in the NIBS group compared to the sham group. In addition, high heterogeneity was observed in the LEMS and 6MWT. Some previous studies have shown that NIBS targeting the motor regions of the lower extremities can activate spinal circuits to improve walking function. However, the evidence of the value of NIBS in improving lower-extremity function in SCI remains limited [17]. In our meta-analysis, the presented results consist of previous studies wherein NIBS showed a significant effect on the lower-extremity strength and balance with the motor cortex selected as the stimulation site. Most of the participants in the analyses were older and in the chronic stage of SCI, which may be associated with a low improvement rate in terms of functional mobility [4]. On the other hand, the effects of NIBS on functional recovery after SCI also depend on the severity of the injury and individuals with a high severity of SCI may have poorer potential for neurological recovery [43]. In total, the fact that one participant with level A and five with level B according to the ASIA grade were included in the analyses may be also associated with poor general improvement in terms of functional mobility in the present study. Therefore, there was no greater improvement in functional mobility in the NIBS group compared to the sham group. In addition, benefits attained with NIBS alone might be confounded by the use of some add-on form of rehabilitation therapy in part [44]. These conventional therapies were not standardized among the studies included in the present review. We also did not account for the differences in form and duration of these add-on therapies in the calculations of efficacy. Hence, high heterogeneity was observed in the LEMS and 6MWT. Furthermore, the results of the mobility analyses may have been influenced by the small number of participants in the primary studies, which is associated with low statistical power and therefore a high probability of type II errors [45].

The present meta-analysis showed that NIBS had a significant benefit in addressing the spasticity of lower limbs evaluated by LMAS, which was consistent with the review performed by Korzhova et al. [46]. However, despite previous studies that have associated the spasticity of the lower extremities with the results of some neurophysiological examinations such as F-waves [22, 35], H reflex, and H/M ratio [22], the H/M ratio in our results failed to show significant change after the NIBS sessions. The results of the H/M ratio analysis may have been affected by the smaller number of studies (only three studies were included). Another equally important cause of the large heterogeneity in the effects of NIBS on spasticity, found in the studies, was the use of different stimulation protocols (frequency, total number of stimuli, stimulation intensity). For example, several TMS studies demonstrated the beneficial effects of theta bursts with a total frequency of 5 Hz in patients with SCI [29]. In other studies of TMS, the effect was also observed for high-frequency stimulation [28, 30]. Therefore, these differences suggest that the potential value of NIBS in spasticity post-SCI requires follow-up in additional in-depth studies.

Although a limited number of clinical trials have shown positive effects of NIBS on upper-extremity motor function in SCI patients, the present study did not find conclusive results of the UMAS, UEMS and JTHFT to support this idea. In line with this, our findings are consistent with the aforementioned studies by Lu et al. and Mateo et al. [47, 48]. The insufficient evidence could be explained by several factors. First, unlike motor dysfunction of the lower extremities, which can occur in the cervical, thoracic, and lumbar levels of SCI, motor dysfunction of the upper extremities only occurs in patients with cervical levels with relatively low incidence. Secord, most of the patients with cervical SCI have severe secondary complications, leading to poorer adherence to a trial’s training specifications [47, 48]. Third, unilateral hemisphere stimulation may not be the most efficacious approach for improving upper-extremity function in SCI, as the motor dysfunction of the upper extremities after SCI is typically bilateral[17]. Fourth, arm and hand function are a complex issue both in SCI and non-SCI patients with tetraplegia, including a wide variety of highly acyclic movements that cannot be easily objectively measured [47, 49].

It should be noted that the differences in demographic indices including age and post-injury time may influence the efficacy of rehabilitation treatments [43, 50, 51]. However, to our surprise, the meta-regression showed that the mean age was not a significant predictor of effect size for the outcome parameters in this review. The first probable explanation for this phenomenon might be the large age range of participants included in each trial. The second explanation may be the small number of studies involved (much less than 10) for each univariate meta-regression analysis, which could affect the results of the meta-regression. The influence of post-injury time on efficacy also has previously been widely investigated [52, 53]. However, the rather limited number of included studies and small sample size in the present study do not allow for firm conclusions to be made from such comparisons between subacute and chronic stages. Further studies directly comparing the different NIBS effects between populations for different ages and stages are necessary for the future.

This review found that some studies reported mild adverse events, such as headaches, facial muscle contraction, and tingling [13, 17, 25, 26, 29]. The most concerning adverse event was a seizure after rTMS [4]. Beyond that, there are no other types of major adverse events were observed in the current review, and no studies reported deterioration in motor function after NIBS. To establish the routine use of NIBS for SCI, it is necessary to develop a method to identify the lowest-risk stimulation parameters. Therefore, we suggest that more clinical evidence is needed in the future regarding the relationship between safety and stimulation parameters in order to improve the effectiveness of treatment.

Overall, our results are important for the emerging field of the use of NIBS in the motor recovery of lower limbs after SCI and support previous findings. Additionally, the present systematic review provides important information for future studies designed to address aspects of motor rehabilitation using NIBS as a rehabilitation tool for individuals after SCI.

### Limitations

Some limitations in the present study should be noted. First, the methodological quality of a few included trials was low, and the study designs differed greatly. Eligibility criteria, random sequence generation, and allocation concealment were heterogeneous or not clearly stated in the articles. The power of the findings and their implications for clinical practice are thereby diminished. Second, our results are restricted to the short-term effects of NIBS, as the included studies did not assess long-term follow-up. Future original research should consider this aspect. Third, we only included articles published in English, which may cause bias if relevant studies have been published in other languages. Fourth, while tDCS and TMS are different types of stimulation with different working mechanisms, our findings indicate that they might trigger comparable effects on motor function. However, we were unable to perform sub-analyses to clarify different types of NIBS techniques and different stimulation parameters due to the small number of studies extracted and the variability in stimulation parameters reported, thus limiting our understanding of the positive changes in motor function promoted by NIBS. Fifth, functional neuroimaging and neurophysiological markers are needed to facilitate a more precise application of NIBS in SCI-related motor dysfunction. Sixth, the forms and parameters of combined rehabilitation therapies during NIBS also varied across the studies. Finally, it remains unclear whether the post-injury time, age, severity of the injury, lesion level, and type of injury are influential factors in NIBS results. These factors should be considered in the formation of homogeneous samples to determine whether these factors are predictors of better motor responses after NIBS.

## Conclusion

From the concept of rehabilitation aimed at improving neuroplasticity, NIBS may be a promising complementary treatment when used in conjunction with conventional therapies or training to enhance motor function in patients with SCI. Our results provide initial evidence of the efficacy of NIBS in improving motor dysfunction in the lower extremities of SCI patients and encourage further high-quality research in this field.

## Supplementary Information

**Additional file 1.** PubMed search strategy.

**Additional file 2.** Univariate meta-regression analysis for NIBS effects based on the mean age.

**Additional file 3.** Sensitivity analysis of the meta-analysis for the effects of NIBS on UMAS, LMA, LEMS, H/M, and 6MWT.

## Data Availability

The datasets supporting the conclusions of this article are included within the article.

## Abbreviations

NIBS: Non-invasive brain stimulation
SCI: Spinal cord injury
SMD: Standardized mean difference
CI: Confidence intervals
rTMS: Repetitive transcranial magnetic stimulation
tDCS: Transcranial direct current stimulation
TBS: Theta-burst stimulation
RCTs: Randomized controlled trials
PRISMA: Preferred Reporting Items for Systematic Reviews and Meta-Analyses
SD: Standard deviation
ASIA: American Spinal Injuries Association
UEMS: Upper Extremity Motor Score
LEMS: Lower Extremity Motor Score
JTHFT: Jebsen Taylor hand function test
10MWT: 10 m walking test
6MWT: 6 m walking test
TUG: Timed up and go test
MAS: Modified Ashworth Scale
H/M: Hmax/Mmax amplitude ratio
BBT: Body balance was measured Berg Balance Test

## References

[1] Duan R, Qu M, Yuan Y, Lin M, Liu T, Huang W (2021). Clinical benefit of rehabilitation training in spinal cord injury: a systematic review and meta-analysis. Spine.

[2] Nam KY, Kim HJ, Kwon BS, Park JW, Lee HJ, Yoo A (2017). Robot-assisted gait training (Lokomat) improves walking function and activity in people with spinal cord injury: a systematic review. J Neuroeng Rehabil.

[3] Gunduz A, Rothwell J, Vidal J, Kumru H (2017). Non-invasive brain stimulation to promote motor and functional recovery following spinal cord injury. Neural Regen Res.

[4] de Araújo AVL, Ribeiro FPG, Massetti T, Potter-Baker KA, Cortes M, Plow EB (2020). Effectiveness of anodal transcranial direct current stimulation to improve muscle strength and motor functionality after incomplete spinal cord injury: a systematic review and meta-analysis. Spinal Cord.

[5] Jo HJ, Perez MA (2020). Corticospinal-motor neuronal plasticity promotes exercise-mediated recovery in humans with spinal cord injury. Brain.

[6] Cofano F, Boido M, Monticelli M, Zenga F, Ducati A, Vercelli A (2019). Mesenchymal stem cells for spinal cord injury: current options, limitations, and future of cell therapy. Int J Mol Sci.

[7] Yi H, Wang Y (2021). A meta-analysis of exosome in the treatment of spinal cord injury. Open Med (Wars).

[8] Li L, Huang H, Yu Y, Jia Y, Liu Z, Shi X (2021). Non-invasive brain stimulation for neuropathic pain after spinal cord injury: a systematic review and network meta-analysis. Front Neurosci.

[9] Kan RLD, Zhang BBB, Zhang JJQ, Kranz GS (2020). Non-invasive brain stimulation for posttraumatic stress disorder: a systematic review and meta-analysis. Transl Psychiatry.

[10] Krogh S, Aagaard P, Jonsson AB, Figlewski K, Kasch H (2022). Effects of repetitive transcranial magnetic stimulation on recovery in lower limb muscle strength and gait function following spinal cord injury: a randomized controlled trial. Spinal Cord.

[11] Yozbatiran N, Keser Z, Davis M, Stampas A, O'Malley MK, Cooper-Hay C (2016). Transcranial direct current stimulation (tDCS) of the primary motor cortex and robot-assisted arm training in chronic incomplete cervical spinal cord injury: a proof of concept sham-randomized clinical study. NeuroRehabilitation.

[12] Kumru H, Murillo N, Benito-Penalva J, Tormos JM, Vidal J (2016). Transcranial direct current stimulation is not effective in the motor strength and gait recovery following motor incomplete spinal cord injury during Lokomat(®) gait training. Neurosci Lett.

[13] Potter-Baker KA, Janini DP, Lin YL, Sankarasubramanian V, Cunningham DA, Varnerin NM (2018). Transcranial direct current stimulation (tDCS) paired with massed practice training to promote adaptive plasticity and motor recovery in chronic incomplete tetraplegia: a pilot study. J Spinal Cord Med.

[14] Horvath JC, Vogrin SJ, Carter O, Cook MJ, Forte JD (2016). Effects of a common transcranial direct current stimulation (tDCS) protocol on motor evoked potentials found to be highly variable within individuals over 9 testing sessions. Exp Brain Res.

[15] Simis M, Fregni F, Battistella LR (2021). Transcranial direct current stimulation combined with robotic training in incomplete spinal cord injury: a randomized, sham-controlled clinical trial. Spinal Cord Ser Cases.

[16] Matheson NA, Shemmell JB, De Ridder D, Reynolds JN (2016). Understanding the effects of repetitive transcranial magnetic stimulation on neuronal circuits. Front Neural Circuits.

[17] Iddings JA, Zarkou A, Field-Fote EC (2021). Noninvasive neuromodulation and rehabilitation to promote functional restoration in persons with spinal cord injury. Curr Opin Neurol.

[18] Higgins J, Green SR. Cochrane Handbook for Systematic Review of Interventions. Version 5.1.0. 2011.

[19] Abdalla MA, Shah N, Deshmukh H, Sahebkar A, Östlundh L, Al-Rifai RH (2022). Impact of pharmacological interventions on insulin resistance in women with polycystic ovary syndrome: a systematic review and meta-analysis of randomized controlled trials. Clin Endocrinol (Oxf).

[20] Alexander MS, Anderson KD, Biering-Sorensen F, Blight AR, Brannon R, Bryce TN (2009). Outcome measures in spinal cord injury: recent assessments and recommendations for future directions. Spinal Cord.

[21] Higgins JP, Thompson SG, Deeks JJ, Altman DG (2003). Measuring inconsistency in meta-analyses. BMJ.

[22] Tolmacheva A, Savolainen S, Kirveskari E, Lioumis P, Kuusela L, Brandstack N (2017). Long-term paired associative stimulation enhances motor output of the tetraplegic hand. J Neurotrauma.

[23] Kumru H, Benito-Penalva J, Valls-Sole J, Murillo N, Tormos JM, Flores C (2016). Placebo-controlled study of rTMS combined with Lokomat((R)) gait training for treatment in subjects with motor incomplete spinal cord injury. Exp Brain Res.

[24] Raithatha R, Carrico C, Powell ES, Westgate PM, Chelette Ii KC, Lee K (2016). Non-invasive brain stimulation and robot-assisted gait training after incomplete spinal cord injury: a randomized pilot study. NeuroRehabilitation.

[25] Benito J, Kumru H, Murillo N, Costa U, Medina J, Tormos JM (2012). Motor and gait improvement in patients with incomplete spinal cord injury induced by high-frequency repetitive transcranial magnetic stimulation. Top Spinal Cord Inj Rehabil.

[26] Gharooni AA, Nair KPS, Hawkins D, Scivill I, Hind D, Hariharan R (2018). Intermittent theta-burst stimulation for upper-limb dysfunction and spasticity in spinal cord injury: a single-blind randomized feasibility study. Spinal Cord.

[27] Gomes-Osman J, Field-Fote EC (2015). Improvements in hand function in adults with chronic tetraplegia following a multiday 10-Hz repetitive transcranial magnetic stimulation intervention combined with repetitive task practice. J Neurol Phys Ther.

[28] Nardone R, Holler Y, Thomschewski A, Brigo F, Orioli A, Holler P (2014). rTMS modulates reciprocal inhibition in patients with traumatic spinal cord injury. Spinal Cord.

[29] Nardone R, Langthaler PB, Orioli A, Holler P, Holler Y, Frey VN (2017). Effects of intermittent theta burst stimulation on spasticity after spinal cord injury. Restor Neurol Neurosci.

[30] Kumru H, Murillo N, Samso JV, Valls-Sole J, Edwards D, Pelayo R (2010). Reduction of spasticity with repetitive transcranial magnetic stimulation in patients with spinal cord injury. Neurorehabil Neural Repair.

[31] Bai Z, Fong KNK, Zhang JJ, Chan J, Ting KH (2020). Immediate and long-term effects of BCI-based rehabilitation of the upper extremity after stroke: a systematic review and meta-analysis. J Neuroeng Rehabil.

[32] Morton RW, Murphy KT, McKellar SR, Schoenfeld BJ, Henselmans M, Helms E (2018). A systematic review, meta-analysis and meta-regression of the effect of protein supplementation on resistance training-induced gains in muscle mass and strength in healthy adults. Br J Sports Med.

[33] Calancie B, Broton JG, Klose KJ, Traad M, Difini J, Ayyar DR (1993). Evidence that alterations in presynaptic inhibition contribute to segmental hypo- and hyperexcitability after spinal cord injury in man. Electroencephalogr Clin Neurophysiol.

[34] Kakulas BA (2004). Neuropathology: the foundation for new treatments in spinal cord injury. Spinal Cord.

[35] Long J, Federico P, Perez MA (2017). A novel cortical target to enhance hand motor output in humans with spinal cord injury. Brain.

[36] Zheng Y, Mao YR, Yuan TF, Xu DS, Cheng LM (2020). Multimodal treatment for spinal cord injury: a sword of neuroregeneration upon neuromodulation. Neural Regen Res.

[37] Serradj N, Agger SF, Hollis ER (2017). Corticospinal circuit plasticity in motor rehabilitation from spinal cord injury. Neurosci Lett.

[38] Fritsch B, Reis J, Martinowich K, Cohen LG, Lu B. Effects of direct currents on long term potentiation in the mouse primary motor cortex in vitro, a possible role of brain derived neurotrophic factor (BDNF). 2008.

[39] Poirrier AL, Nyssen Y, Scholtes F, Multon S, Rinkin C, Weber G (2004). Repetitive transcranial magnetic stimulation improves open field locomotor recovery after low but not high thoracic spinal cord compression-injury in adult rats. J Neurosci Res.

[40] Gao W, Yu LG, Liu YL, Wang YZ, Huang XL (2015). Mechanism of GABA receptors involved in spasticity inhibition induced by transcranial magnetic stimulation following spinal cord injury. J Huazhong Univ Sci Technolog Med Sci.

[41] Gao W, Yu LG, Liu YL, Chen M, Wang YZ, Huang XL (2017). Effects of high frequency repetitive transcranial magnetic stimulation on KCC2 expression in rats with spasticity following spinal cord injury. J Huazhong Univ Sci Technolog Med Sci.

[42] Nardone R, Holler Y, Brigo F, Orioli A, Tezzon F, Schwenker K (2015). Descending motor pathways and cortical physiology after spinal cord injury assessed by transcranial magnetic stimulation: a systematic review. Brain Res.

[43] Richard-Denis A, Chatta R, Thompson C, Mac-Thiong JM (2020). Patterns and predictors of functional recovery from the subacute to the chronic phase following a traumatic spinal cord injury: a prospective study. Spinal Cord.

[44] Li Y, Fan J, Yang J, He C, Li S (2018). Effects of transcranial direct current stimulation on walking ability after stroke: a systematic review and meta-analysis. Restor Neurol Neurosci.

[45] Borenstein M, Hedges LV, Higgins JP, Rothstein HR (2010). A basic introduction to fixed-effect and random-effects models for meta-analysis. Res Synth Methods.

[46] Korzhova J, Sinitsyn D, Chervyakov A, Poydasheva A, Zakharova M, Suponeva N (2018). Transcranial and spinal cord magnetic stimulation in treatment of spasticity: a literature review and meta-analysis. Eur J Phys Rehabil Med.

[47] Lu X, Battistuzzo CR, Zoghi M, Galea MP (2015). Effects of training on upper limb function after cervical spinal cord injury: a systematic review. Clin Rehabil.

[48] Mateo S, Di Marco J, Cucherat M, Gueyffier F, Rode G (2020). Inconclusive efficacy of intervention on upper-limb function after tetraplegia: a systematic review and meta-analysis. Ann Phys Rehabil Med.

[49] O'Brien AT, Bertolucci F, Torrealba-Acosta G, Huerta R, Fregni F, Thibaut A (2018). Non-invasive brain stimulation for fine motor improvement after stroke: a meta-analysis. Eur J Neurol.

[50] Tedla JS, Dixit S, Gular K, Abohashrh M (2019). Robotic-assisted gait training effect on function and gait speed in subacute and chronic stroke population: a systematic review and meta-analysis of randomized controlled trials. Eur Neurol.

[51] Marzolini S, Wu CY, Hussein R, Xiong LY, Kangatharan S, Peni A (2021). Associations between time after stroke and exercise training outcomes: a meta-regression analysis. J Am Heart Assoc.

[52] Luo L, Meng H, Wang Z, Zhu S, Yuan S, Wang Y (2020). Effect of high-intensity exercise on cardiorespiratory fitness in stroke survivors: a systematic review and meta-analysis. Ann Phys Rehabil Med.

[53] Mahmood A, Veluswamy SK, Hombali A, Mullick A, Manikandan N, Solomon JM (2019). Effect of transcutaneous electrical nerve stimulation on spasticity in adults with stroke: a systematic review and meta-analysis. Arch Phys Med Rehabil.

