# Supplementary material for: Effects of non-invasive brain stimulation on motor function after spinal cord injury: a systematic review and meta-analysis
Source: J Neuroeng Rehabil. 2023 Jan 12;20:3. doi: 10.1186/s12984-023-01129-4 (PMC9837916; doi:10.1186/s12984-023-01129-4)
Supplement: Supplementary file 1 — Additional file 1. PubMed search strategy. [file 12984_2023_1129_MOESM1_ESM.docx]

**PubMed search strategy:**

**Search:** ((("Spinal Cord Injuries"[MeSH Terms] OR "Spinal Fractures"[MeSH Terms] OR "Spinal Cord Ischemia"[MeSH Terms] OR "Paraplegia"[MeSH Terms] OR "Quadriplegia"[MeSH Terms] OR "SCI"[Title/Abstract] OR "spinal cord"[Title/Abstract]) AND (("Transcranial Magnetic Stimulation"[Mesh]) OR ("Transcranial Direct Current Stimulation"[Mesh]) OR ("transcranial magnetic stimulation") OR (" rtms") OR ("TMS") OR ("non-invasive brain stimulation") OR ("motor cortex excitability") OR ("deep brain stimulation") OR ("theta burst stimulat*") OR ("TBS") OR ((magnetic or brain or non-invasive or noninvasive or corticospinal-motoneuronal) AND (stimulation or neuromodulation)) OR ("Transcranial Electrical") OR ("tDCS*") OR(" tCS"))) AND ((Controlled Clinical Trial) OR (randomized))) NOT ((pain[Title/Abstract]) OR (bladder [Title/Abstract]))
