# Supplementary material for: Effects of non-invasive brain stimulation on motor function after spinal cord injury: a systematic review and meta-analysis
Source: J Neuroeng Rehabil. 2023 Jan 12;20:3. doi: 10.1186/s12984-023-01129-4 (PMC9837916; doi:10.1186/s12984-023-01129-4)
Supplement: Supplementary file 2 — Additional file 2. Univariate meta-regression analysis for NIBS effects based on the mean age. [file 12984_2023_1129_MOESM2_ESM.pdf]

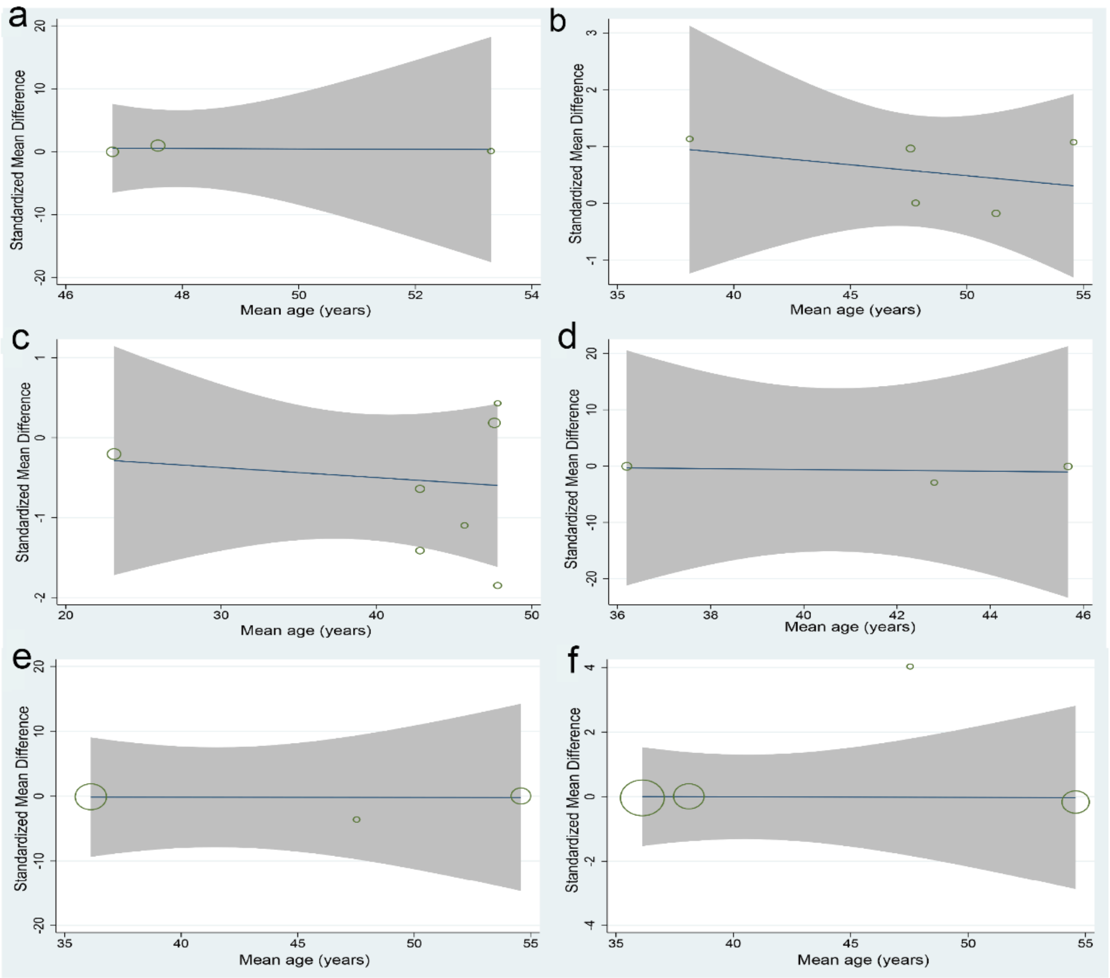

Fig.S1 Meta-regression of effect size (standardized mean difference) by mean age of participant. (a) UEMS: β =0.960, standard error =0.177, *P* =0.862; (b) LEMS: β =0.967, standard error =0.057, *P* =0.602; (c) LMAS: β =0.985, standard error =0.038, *P* =0.712; (d) H/M: β =-0.079, standard error =0.329, *P* =0.851; (e) 6MWT: β =-0.020, standard error =0.201, *P* =0.937; (f)TUG: β =0.998, standard error =0.042, *P* =0.972.
