# Supplementary material for: Effects of non-invasive brain stimulation on motor function after spinal cord injury: a systematic review and meta-analysis
Source: J Neuroeng Rehabil. 2023 Jan 12;20:3. doi: 10.1186/s12984-023-01129-4 (PMC9837916; doi:10.1186/s12984-023-01129-4)
Supplement: Supplementary file 3 — Additional file 3. Sensitivity analysis of the meta-analysis for the effects of NIBS on UMAS, LMA, LEMS, H/M, and 6MWT. [file 12984_2023_1129_MOESM3_ESM.pdf]

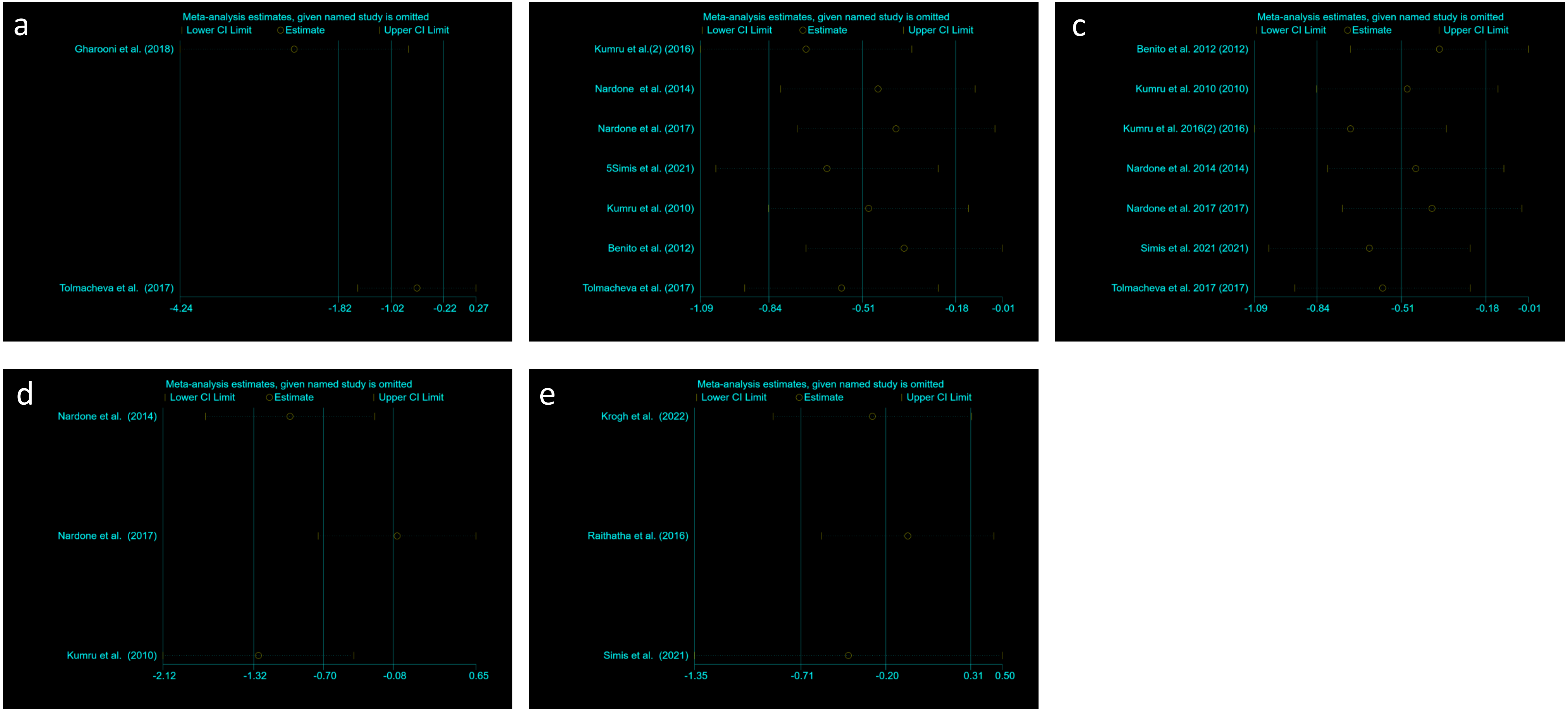

Fig.S2 Sensitivity analysis of the meta-analysis for the effects of NIBS on (a) UMAS, (b) LMAS, (c) LEMS, (d) H/M and (e) 6MWT.
